# A prospective investigation into the effect of colchicine on tuberculous pericarditis

**DOI:** 10.5830/CVJA-2016-035

**Published:** 2016

**Authors:** Jurgens Liebenberg, Catherine Jane Dold, Lourens Rasmus Olivier

**Affiliations:** Worcester Hospital, Worcester, South Africa; Hanover Park Day Hospital, Cape Town, South Africa; Medi-Clinic Hospital, Durbanville, South Africa

**Keywords:** pericarditis, colchicine, tuberculosis, constrictive pericarditis

## Abstract

**Introduction:**

Tuberculous (TB) pericarditis carries significant mortality and morbidity rates, not only during the primary infection, but also as part of the granulomatous scar-forming fibrocalcific constrictive pericarditis so commonly associated with this disease. Numerous therapies have previously been investigated as adjuvant strategies in the prevention of pericardial constriction. Colchicine is well described in the treatment of various aetiologies of pericarditis. The aim of this research was to investigate the merit for the use of colchicine in the management of tuberculous pericarditis, specifically to prevent constrictive pericarditis.

**Methods:**

This pilot study was designed as a prospective, double-blinded, randomised, control cohort study and was conducted at a secondary level hospital in the Northern Cape of South Africa between August 2013 and December 2015. Patients with a probable or definite diagnosis of TB pericarditis were included (n = 33). Study participants with pericardial effusions amenable to pericardiocentesis underwent aspiration until dryness. All patients were treated with standard TB treatment and corticosteroids in accordance with the South African Tuberculosis Treatment Guidelines. Patients were randomised to an intervention and control group using a webbased computer system that ensured assignment concealment. The intervention group received colchicine 1.0 mg per day for six weeks and the control group received a placebo for the same period. Patients were followed up with serial echocardiography for 16 weeks. The primary outcome assessed was the development of pericardial constriction. Upon completion of the research period, the blinding was unveiled and data were presented for statistical analysis.

**Results:**

TB pericarditis was found exclusively in HIV-positive individuals. The incidence of pericardial constriction in our cohort was 23.8%. No demonstrable benefit with the use of colchicine was found in terms of prevention of pericardial constriction (p = 0.88, relative risk 1.07, 95% CI: 0.46–2.46). Interestingly, pericardiocentesis appeared to decrease the incidence of pericardial constriction.

**Conclusion:**

Based on this research, the use of colchicine in TB pericarditis cannot be advised. Adjuvant therapy in the prevention of pericardial constriction is still being investigated and routine pericardiocentesis may prove to be beneficial in this regard.

## Introduction

South Africa, a land of stark contrasts, contains a diverse natural beauty that can easily be compared with some of the world’s most majestic outdoor scenes. One of the new seven wonders of the natural world, Table Mountain, parades its splendour to the capital of South Africa, Cape Town. Unfortunately, South Africa is also considered by many to be one of the tuberculosis (TB) capitals of the world. The incidence of TB in South Africa is estimated to have increased by over 400% in the past 15 years. This is confounded by a staggering co-infection rate of approximately 73% with the human immunodeficiency virus (HIV).^1^

One of the most dreaded complications of TB pericarditis is pericardial scar formation. Due to scarring, the pericardium becomes calcified and contracts over the cardiac chambers, thereby encasing the heart in a fibrocalcific skin that impedes diastolic filling.^2^ Constrictive pericarditis (CP) is the natural consequence of about 17 to 40% of cases of TB pericardial infection.^3^ The definitive treatment of CP is surgical removal of the pericardium, a procedure with a significant peri-operative mortality rate of approximately 15%.^4^

South Africa is on the forefront of research on TB heart disease and has recently published the large, multi-centre IMPI trial.^5^ One of the goals of the IMPI trial was to assess the impact of corticosteroids in the management of TB pericarditis. The major findings of the study included (1) corticosteroids had no impact on mortality rates in patients with TB pericarditis, (2) corticosteroids decreased the incidence of pericardial constriction by 46%, and (3) HIV-positive patients who received corticosteroids had a significantly increased risk of developing HIV-associated malignancies.

In established TB, early and effective treatment with shortcourse anti-TB therapy is the mainstay of management. Various strategies have been investigated as adjuncts to anti-TB drugs in the prevention of pericardial constriction. The ongoing discussions and numerous investigations into a wide array of agents as possible ‘magic bullets’ in the prevention of pericardial constriction (post-TB infection) illustrates both the interest in the field, and also the lack of a satisfying solution to this problem. The following strategies have previously been evaluated: Mycobacterium indicus pranii immunotherapy,^5^ corticosteroids,^5^ pericardiocentesis,6 open surgical drainage (pericardial window),^7^ thalidomide,^8^ instilling intrapericardial fibrinolytic therapies,^9^-^11^ and a wide array of non-steroidal anti-inflammatory medication. Not one of these therapies has, to date, been internationally recognised as an acceptable standard of therapy, and the choice of adjuvant treatment varies significantly among experts in the field.

Colchicine is an inhibitor of microtubule polymerisation. It acts by binding to tubulin and is registered for the acute treatment of gout crystal arthropathies. The plant source of colchicine, the autumn crocus (Colchicum autumnale), was described as treatment for arthritis in the Ebers Papyrus in 1500 BC.^12^ In modern medicine, colchicine has however played a wider role in the treatment of pericarditis of various aetiologies, both acute and chronic. This has been investigated in a prospective, randomised trial named COPE (Colchicine for Acute Pericarditis),13 and the major findings concluded that colchicine significantly reduced the recurrence rates and symptom persistence due to pericarditis. To date however, the use of colchicine has, to the best of our knowledge, never been systematically assessed in the context of pericardial TB. The purpose of this research was to assess the merit for the use of colchicine in the context of TB pericarditis.

## Methods

This research was conducted in the Northern Cape province of South Africa at a secondary-level hospital in Kimberley between August 2013 and April 2015. The research was approved by the ethics committee of the University of the Free State and the study was registered with the National Health Research Committee. The research was conducted in accordance with the Declaration of Helsinki.

This pilot study was designed as a prospective, doubleblind, randomised, control cohort. All patients presenting to the Kimberley Hospital complex (KHC) with pericardial effusions were assessed for inclusion and exclusion criteria. In the absence of contra-indications, patients underwent therapeutic pericardiocentesis if the procedure was deemed safe and possible. Standard therapy was initiated in accordance with the South African National Tuberculosis Management Guidelines:14 weight-adjusted anti-TB drugs (Rifafourf^®^) and oral corticosteroids. (prednisone: 1.5 mg/kg per day for four weeks; 1.0 mg/kg per day for two weeks; 0.5 mg/kg per day for one week; 0.25 mg/kg per day for one week). HIV co-infected patients not previously on treatment were initiated on fixeddose combination (FDC) antiretroviral treatment six weeks after initiation of TB treatment (FDC: Tenofovir Disoproxil Fumarate 300 mg, Emtricitabine 200 mg and Efavirenz 600 mg).

**Fig. 1.**
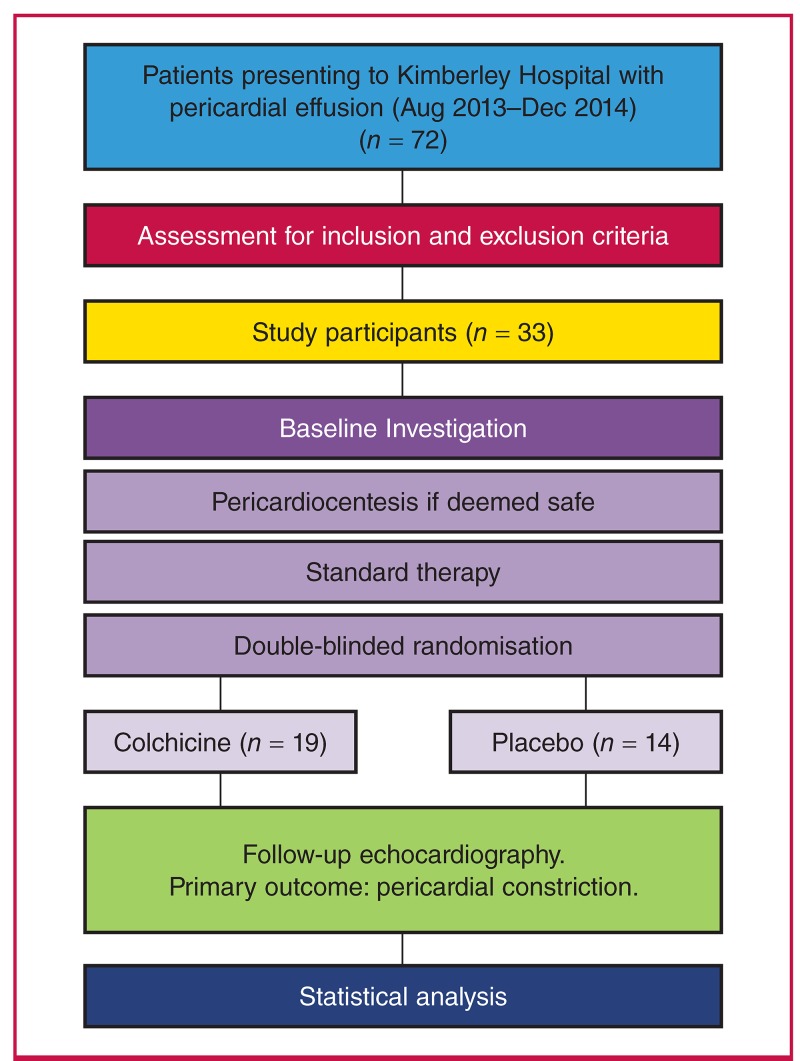
Flow diagram illustrating study methodology.

Patients were randomly assigned to the intervention group with the use of a web-based randomisation system that ensured assignment concealment. The intervention group received colchicine (dose 1.0 mg per day) for a total of six weeks, whereas the control group received a placebo for the same period (Fig. 1).

Patients subsequently underwent serial echocardiographic examinations on an out-patient basis and adherence checks, including pill counts, were done at follow-up visits. The primary outcome assessed was the development of pericardial constriction and this diagnosis was made echocardiographically at four months post initial presentation. Upon completion of the follow-up period of all patients, the blinding was unveiled and data were presented for statistical analysis.

Two groups of patients were included: (1) definite TB pericarditis: the presence of TB bacilli was observed on microscopic examination of pericardial fluid; cultures of pericardial fluid were positive for Rifampicin-sensitive Mycobacterium tuberculosis (MTB); pericardial fluid was positive for MTB on direct polymerase chain reaction (PCR) (Gene Xpert); and (2) probable TB pericarditis: proof of TB was found elsewhere (positive cultures for MTB on sputum or cerebrospinal fluid); pericardial fluid with adenine deaminase (ADA) level > 40 U/l; a total diagnostic index score > 6 on using the Tygerberg clinical prediction score (Table 1).^15^

**Table 1:** The Tygerberg clinical prediction score for the diagnosis of TB pericarditis. A total diagnostic score > 6 yields a sensitivity of 82% and a specificity of 76% for the diagnosis of TB pericarditis

*Admission variable*	*Diagnostic index*
Weight loss	1
Night sweats	1
Fever	2
Serum globulin > 40 g/l	3
Leukocyte count < 10 × 109	3

The exclusion criteria were: patients with renal or hepatic impairment (creatinine clearance rate > 85 ml/min or transaminases > 1.5 upper limit of normal); and pregnant patients or patients intending to become pregnant within four months.

The gold-standard diagnostic test for the diagnosis of CP is the demonstration of increased interventricular interdependence during cardiac catheterisation. Doppler echocardiography and other novel echocardiographic techniques have provided us with reliable non-invasive alternatives to the diagnosis of CP. In this study, the diagnosis of CP was made by means of echocardiography by adhering to the principles in the article by Dal-Bianco et al. on the echocardiographic diagnosis of CP.^16^

Initial echocardiographic assessment ensured that no features of constriction were present at the time of enrolment in the study. Follow-up echocardiograms were performed four months after the initiation of therapy.

**Fig. 2.**
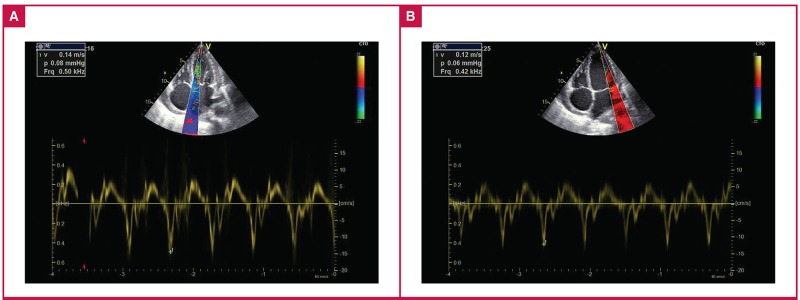
A. Tissue Doppler of the medial aspect of the mitral valve annulus demonstrating early diastolic tissue velocity of 0.14 m/s. B. Tissue Doppler of the lateral aspect of the mitral valve annulus showing early diastolic tissue velocity of 0.12 m/s. The lower tissue velocity on the lateral aspect is the opposite of the normal phenomenon (annulus reversus).

**Fig. 3.**
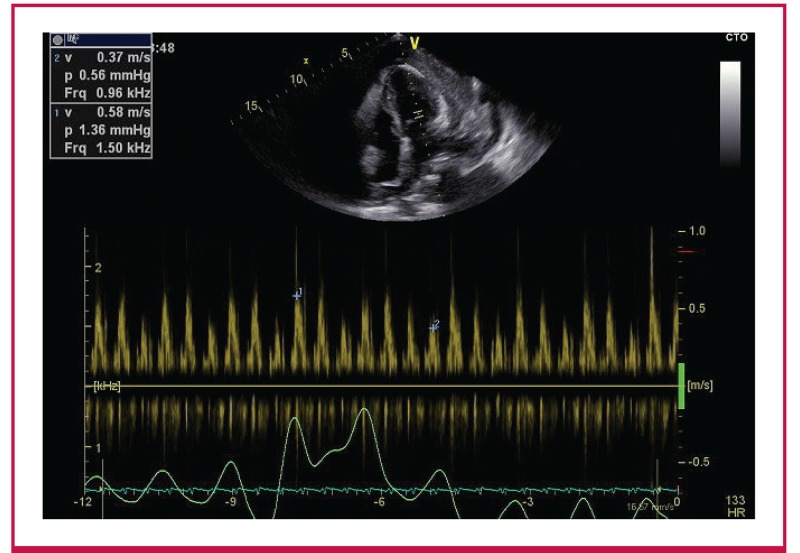
Pulse-wave Doppler at the level of the mitral valve leaflet tips demonstrating a respiratophasic variation in the early diastolic transmitral inflow velocities in excess of 25%.

**Fig. 4.**
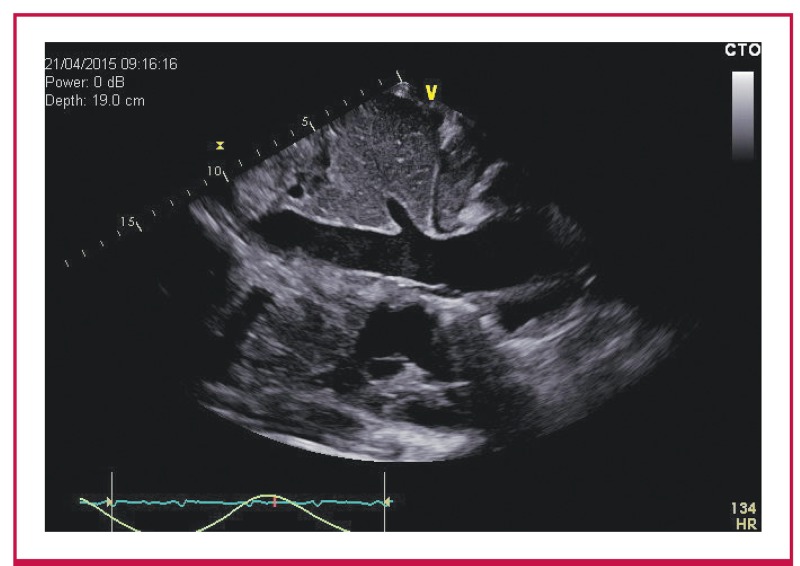
Dilated and distended inferior vena cava. No respiratory variation was observed.

The echocardiograms were performed and co-reviewed by two experienced echocardiographers (who had both attended a dedicated workshop at a tertiary-level academic hospital aimed at the echocardiographic diagnosis of CP). A GE Vivid E6® ultrasound machine was used to perform a systematic examination according to the basic minimum standards as stipulated by the British Society of Echocardiography.^17^ Numerous other echocardiographic parameters were assessed, including the presence of a septal shudder, respiratophasic septal shift, left atrial enlargement and echocardiographic features of pericardial thickening (Figs 2–4).

## Statistical analysis

Statistical analysis was performed by the Department of Biostatistics of the University of the Free State, Bloemfontein, South Africa. The SAS Version 8.3 was used. Groups were compared regarding outcomes using frequency tables with appropriate hypothesis testing (chi-squared of Fisher’s exact test) and 95% confidence intervals for differences in percentages. The standard deviation value p < 0.05 was considered significant.

## Results

**Fig. 5.**
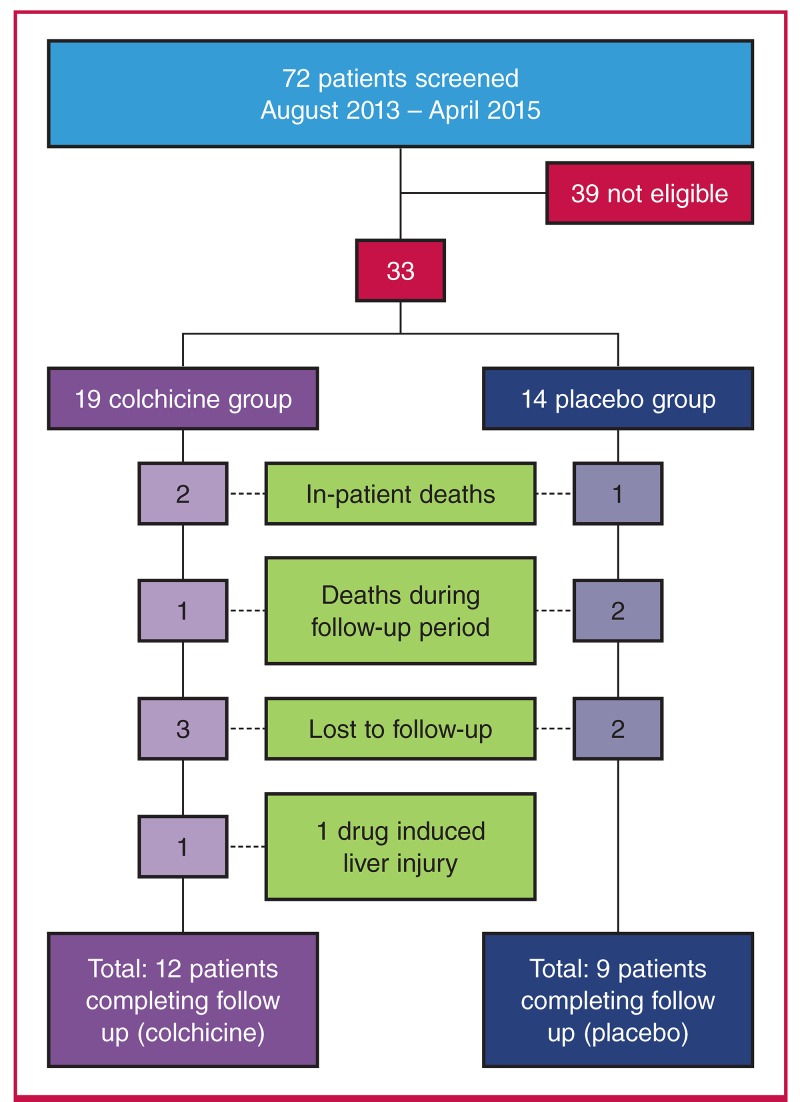
Screening, randomisation, follow up and analysis of the study patients.

Thirty-three patients met the initial inclusion criteria. Three patients passed away while in hospital and an additional three passed away during the follow-up period. In-patient deaths were due to neutropenic sepsis, cerebrovascular incident and nosocomial pneumonia, respectively. In all out-patient deaths, the cause was undetermined. Five patients were lost to follow up and one patient was removed from the study due to presumed drug side effects. A total of 21 patients completed the follow-up period (Fig. 5).

The study population had a female preponderance (66% females) and the mean age of the studied patients was 31 years. Disseminated pericardial tuberculosis was found to be a disease exclusive to the immune-compromised in this cohort; all 21 patients were HIV positive. The median CD4^+^ count was 162 and 346 cells/mm^3^ in the colchicine and placebo groups, respectively.

Of the 21 eligible participants, 12 had been assigned to the treatment group and the remaining nine were in the placebo group. The diagnosis of definite pericardial tuberculosis was made in 23.8% of the patients, while the remaining 76.2% were diagnosed on the basis of suggestive clinical and biochemical features (see inclusion criteria). Of the studied patients, 47.6% underwent pericardiocentesis, whereas the remaining 52.4% could not undergo safe pericardiocentesis.

The average volume of fluid drained via single pericardial aspiration was 622 ml. The macroscopic appearance of the fluid varied from serosanguineous to haemorrhagic, reflecting the different pathological stages of development. Mycobacterium tuberculosis was proven on pericardial aspirates in 50% of cases, either by positive culture (30%) or by direct PCR technique (Gene Xpert) (20%) (Table 2).

**Table 2:** Pericardial fluid biochemistry

*Biochemical parameter*	*Average*
Protein (g/l)	62.7
ADA (U/l)	96.6
LDH (U/l)	4494
pH	7.3
Glucose (mmol/l)	2.8

Pericardial constriction is the natural sequela of approximately 17 to 40% of TB pericardial infections.^3^ In our cohort, the incidence of pericardial constriction (demonstrated by echocardiography) four months after the initial diagnosis was 23.8%. Of the five patients who developed pericardial constriction, two were in the control group and the remaining three were in the group treated with colchicine. Of those who did not develop pericardial constriction, nine were in the colchicine group and seven were in the placebo group.

The data from Table 3 yields a p-value of 0.88. The relative risk for developing constriction in the colchicine group compared to the intervention group was 1.07 (95% CI: 0.46–2.46). There was therefore no statistically demonstrable correlation between the use of colchicine and pericardial constriction in this study cohort.

**Table 3:** Two-by-two table demonstrating the primary study outcome

**	*Colchicine*	*Placebo*	*Total*
Constriction	3	2	5
No constriction	9	7	16
Total	12	9	21

The side effects among the patients using colchicine were usually minor; 56% of the initial 19 patients who were in the colchicine group reported self-limiting diarrhoea during their hospital stay. Serious side effects were observed in one patient who developed hepatitis during his course of treatment. The patient was removed from the study and daily liver function testing showed a rapid recovery.

Although the study was neither empowered nor designed to evaluate the effect of pericardiocentesis on the subsequent development of pericardial constriction, a very apparent and interesting finding was observed. We found that, with the exception of one patient, all those who developed pericardial constriction were in the group that did not undergo pericardiocentesis. Conversely, in the group that underwent pericardiocentesis, only one participant developed pericardial constriction. Pericardiocentesis therefore seemed to be very effective in the prevention of pericardial constriction and in this cohort only one patient (10%) who underwent pericardiocentesis developed constriction. These findings are observational and disregard the initial group allocations.

## Discussion

The proverbial ‘eureka moment’ in the management of TB pericarditis seems to be elusive. Numerous interventions have been postulated and investigated in an attempt to prevent the devastating post-inflammatory changes in the pericardium following TB pericarditis. In this pilot study, the merit of adding colchicine to the current management guidelines was investigated in a systematic manner. As all the participants of this study were HIV positive, the findings can only be applied to this subgroup of patients with TB pericarditis.

There was a notable difference in the median CD4^+^ lymphocyte count between the treatment and placebo groups, but when assessed as an independent variable, no correlation could be demonstrated between degree of immunocompetency, as measured by CD4^+^ count, and the risk for development of constriction.

This pilot study could not demonstrate any benefit derived from the addition of colchicine to the routine management of HIV-positive patients with TB pericarditis. The power of this pilot trial was insufficient to detect small differences in outcome; however, it appears that colchicine use has no correlation with the prevention or formation of post-TB CP. This pilot trial could not assess the beneficial effects of colchicine in the HIV-negative patient with TB pericarditis.

After considering the findings of this pilot research, the costs of the drug, the polypharmacy these patients are exposed to, drug–drug interactions and side effects (albeit mild), this study would advise against the use of colchicine in the management of HIV-positive patients with TB pericarditis.

The implementation of a pericardiocentesis until dryness (with or without extended drainage) was up to this point never studied in a controlled or comparative manner. Research conducted by Reuter et al.^6^ in 2007 found the first evidence to suggest the benefit of a pericardiocentesis until dryness with extended drainage. In their research, 162 patients with TB pericarditis underwent pericardiocentesis, and over a followup period of six years, only two patients (1.23%) developed fibrous pericardial constriction. The research concluded that echocardiographic-guided pericardiocentesis with extended drainage is a safe and effective management option, and when combined with short-course anti-tuberculous therapy, it almost completely prevents the development of CP.

A few leading centres are employing a routine ‘pericardiocentesis until dryness’ approach based on this literature, whereas most do not. The interesting observation made in our pilot study was that the findings made by Reuters et al. in 2007 were reproducible on a much smaller scale. Pericardial constriction, although having a low incidence, was almost exclusively seen in the group that did not undergo pericardiocentesis (observational – disregard original group allocation). As suggested by some expert opinion and as supported by the data published by Reuters et al. and observational findings of our pilot trial, the practice of routine pericardiocentesis until dryness in the absence of contraindications appears to be the preferred management option and this might well be the long-awaited ‘eureka moment’, in an attempt to halt the development of pericardial constriction.

## Linitations of the study

The diagnosis of pericardial constriction was made with echocardiography, whereas the gold standard for diagnosing CP is invasive haemodynamic studies. Work done by Oh et al.^18^ and Boonyaratevej et al.^19^ demonstrated that one of the most characteristic findings of CP, a respiratory variation in early transmittal inflow velocity, is neither perfect in its sensitivity nor specificity for the diagnosis. In patients with markedly elevated left atrial pressures, the respiratory variation in the inflow velocities may be less than 25%. Furthermore, in patients with chronic obstructive pulmonary disease and severe right ventricular dysfunction, the variation may be elevated in the absence of CP. This research emphasises the importance of using a variety of recognised echocardiographic diagnostic tools to confirm a non-invasive diagnosis of CP.

The duration of follow up was only four months. Some comparative research had follow-up periods of up to six years. Most patients who develop CP, do so in a period of three to four months. There may however be patients who will only develop constriction after four months. Research to address this aspect may be valuable.

Corticosteroids were used as part of the standard therapy in all patients. However, subsequent to the initiation of the research, the IMPI trial brought to light their findings that corticosteroids should not be used in TB pericarditis in HIV-infected patients. The South African National TB guidelines published in 2014 still advised the use of corticosteroids in all patients and the findings of the IMPI trial had not yet been incorporated into current South African National Tuberculosis Management Guidelines.^15^

## Conclusion

Based on current research, the use of colchicine in addition to standard antituberculous therapy cannot be advised in the context of TB pericarditis in the HIV-positive population. The jury is still out on which adjuvant strategies may prove to be beneficial in the prevention of CP, especially in the HIV-coinfected subgroup. Based on observations from this research and some other studies, routine pericardiocentesis until dryness with extended drainage may prove to be the long-awaited solution to the common dilemma of post-TB CP.
